# Differential Effects of Oxytocin on Visual Perspective Taking for Men and Women

**DOI:** 10.3389/fnbeh.2017.00228

**Published:** 2017-11-15

**Authors:** Tong Yue, Yuhan Jiang, Caizhen Yue, Xiting Huang

**Affiliations:** ^1^Faculty of Psychology, Southwest University, Chongqing, China; ^2^Post-doctoral Station of Mathematics, Southwest University, Chongqing, China; ^3^School of Humanities, Shandong Management University, Jinan, China; ^4^Department of Education, Chongqing University of Arts and Sciences, Chongqing, China

**Keywords:** oxytocin, theory of mind, perspective taking, egocentric biases, sex differences

## Abstract

Although oxytocin (OXT) has been shown to lead to reduced self-orientation, no study to date has directly and effectively weakened the egocentric tendencies in perspective taking tasks for both men and women. In this double-blind, placebo-controlled, mixed design study we investigated the effects of OXT on men and women in visual perspective taking tasks. The results showed that OXT shortened the differences in response time between men and women in all experimental conditions. In addition, after OXT administration, the difference in reaction time between judging from one's own perspective and judging from others' perspectives decreased in female participants; however, this effect was not present in males. This may indicate that under OXT treatment, women have a higher tendency to overcome interference from their position and mindset when judging others' perspectives. However, OXT did not affect participants' accuracy, which is possibility because the used task was not suited to detect performance improvements caused by OXT. In summary, the above results may indicate that OXT could increase perspective-taking abilities through reducing self-bias and increasing the perception of others; furthermore, this trend mainly affected women rather than men.

## Introduction

Perspective taking is the psychological process of contemplating and inferring other perspectives (Galinsky et al., 2005). The essential characteristic of the process is to set aside one's own perspective in order to see through the others' eyes, to imagine what others might think or feel, or to achieve what is sometimes colloquially referred to as “putting oneself in another's shoes.” However, previous research has shown that resisting interference from one's own perspective is not easily achieved. For example, children under the age of four cannot distinguish their own mental state from others; in the false-belief task, they often respond according to their own mental state (Moore et al., 1995; Wellman et al., 2001). Even when adults reason about others' beliefs or thinking, egocentric biases are common (Keysar et al., 2003; Royzman et al., 2003; Bernstein et al., 2004; Apperly et al., 2009), particularly when under cognitive load (Epley et al., 2004). Many researchers believe that self-centeredness is a default choice when inferring other people's mental states (Decety and Sommerville, 2003), although this bias could provide a reasonable starting point and reference for understanding others' mental states (Epley, 2008). However, self-centeredness sometimes renders people unable to distinguish between themselves and others effectively, which results in difficulties in communication and interaction (Keysar et al., 2000) and thus often requires correction or constraint when attempting to adopt someone else's perspective.

The effects of oxytocin (OXT) have become a major focus of research in modern biological psychology (Heinrichs and Domes, 2008; Heinrichs et al., 2009; Meyer-Lindenberg et al., 2011; Kumsta and Heinrichs, 2013). While there is ongoing debate concerning the precise nature and mechanisms of the effects of OXT in humans, it is generally considered that it may primarily operate as an enhancement of the salience of social stimuli and affiliative behaviors (Bartz et al., 2011; Shamay-Tsoory and Abu-Akel, 2016). In recent years, interest has also been increasing in its potential role in influencing the ability of perspective taking. Intranasal dosing of OXT, which is believed to cross the blood–brain barrier and achieve access to the CNS (Neumann et al., 2013; Striepens et al., 2013), has been found to increase the ability of perspective taking. From the perspective of strategic judgments, Domes et al. (2007) reported that OXT could improve the ability to infer the mental state of others from the eye region. Aydogan et al. (2017) further reported that participants who received OXT were significantly better at predicting the actions of others, which indicated that OXT could enhance perspective taking in strategic interactions. Moreover, Shamay-Tsoory et al. (2013) reported that intranasal OXT led to a remarkable increase in empathy for the pain of even adversary out-group members, demonstrating its important role for promoting perspective taking in emotional judgments.

In fact, the positive effect of OXT on perspective taking may be related to its potential role in influencing aspects of self-processing and in particular distinctions between self and other. Colonnello et al. (2013) reported that OXT reduced the threshold to distinguish between one's own face and an unfamiliar face in a morphing paradigm, indicating its role in sharpening the self-other perceptual boundary. A study about the empathy for pain reported that OXT only increased the empathy for pain ratings toward others when participants had been instructed to adopt the perspective of another, but not when they adopted a self-perspective (Abu-Akel et al., 2015). Further study also reported that OXT could reduce the sense of agency in anxiously attached individuals, indicating OXT's effects of reduced self-orientation (Bartz et al., 2015). In addition, OXT's established role in promoting affiliative behavior and social bonds (Bartz et al., 2011; Striepens et al., 2011; Bethlehem et al., 2013) seems consistent with results reporting a decrease in self-interest and an increase in interest in others. Considering these characteristics of OXT raises the question of whether it can effectively weaken egocentric tendencies in perspective taking. We investigated this question in our study, which, to our knowledge, has not yet been explored.

Our discussion of the problem is aided by the basic visual perspective taking process, as a typical paradigm through which to explore the cognitive process of distinction between the self and others. The experimental paradigm we used in this study originates from a study by Samson et al. (2010), where participants were presented with a picture of a room in which either one or two walls displayed red discs. A human avatar stands facing one of the walls on which red discs are displayed. During the consistent perspective condition, both the participant and the avatar could see the same number of discs. However, in the inconsistent perspective condition, the participant and the avatar saw a different number of discs (some of the discs were not visible to the avatar). Participants were then asked to identify whether they were able to see the same number of discs as the avatar. Perhaps, due to interference by the ego in perspective taking, many studies found that, in the inconsistent conditions, judging from the perspective of the avatar resulted in slower response times and more errors compared to when participants judged from their own perspective, which is a typical example of egocentric bias (Samson et al., 2010; Wang et al., 2015). On this basis, we speculate that, if OXT decreased self-centeredness, it may also affect subjects in the visual perspective taking task; i.e., by reducing egocentric bias.

In addition, differences in sex are an important factor when examining the effects of OXT on human social cognition. Many previous research results, including social judgment (Hoge et al., 2014; Gao et al., 2016), social approach/avoidance (Theodoridou et al., 2013; Preckel et al., 2014), social cooperation/competition (Fischer-Shofty et al., 2013; Scheele et al., 2014), and the ability to maintain social relations (Yao et al., 2014) have found inconsistent and even opposing results in the effects of OXT in different genders. Currently however, there is no definite explanation for why such gender differences exist and it is difficult to predict under what type of social situation the effects of OXT will cause such a difference. Following this, in our exploration of the effects of OXT on the visual perspective taking task, we wondered whether this study could also provide insight into differences between males and females. To address this question, both men and women were recruited and the results were compared and analyzed to explore the role of OXT in the information processing system of self and others and to examine potential differences in results across male and female participants.

## Methods

### Participants and treatment

Subjects from the Southwest University and the Chongqing University of Arts and Sciences, in China, were recruited through local advertisements. Each subject was provided with a written informed consent form prior to study enrollment. Eighty-five students (39 males and 46 females, with a mean age of 21.2 years; S.D. = 1.76) participated in the study. None of the subjects were taking any form of medication or reported having had neurological problems or psychiatric illnesses prior to the start of the study. None of the female subjects were menstruating, which is important because the menstrual cycle may influence the effectiveness of OXT administration (Bakermans-Kranenburg and van IJzendoorn, 2013) and no women were pregnant or using oral contraceptives. Before the formal experiment, we asked the participants to maintain their regular sleep pattern and abstain from caffeine, alcohol, and smoking for at least 12 h prior to the experiment. After a detailed explanation of the study protocol, all subjects were asked to sign a written informed consent form. The study was approved by the Ethics Committee of the Southwest University and the Chongqing University of Arts and Sciences, and all involved procedures were in accordance with the sixth revision of the Declaration of Helsinki.

The study used a double-blind, placebo-controlled, mixed design. In the experiment, all subjects first received a single intranasal dose of 24 IU OXT (Syntocinon Spray, Sichuan Meike Pharmacy Co. Ltd, China; three puffs of 4 IU per nostril with 30 s between each puff) or PLC (with the identical type of bottle from the same pharmaceutical company, containing all of the same ingredients as the OXT nasal spray except the neuropeptide, i.e., sodium chloride and glycerin; also, three puffs were administered per nostril). In line with a previous study (Striepens et al., 2011), the formal experiments started 45 min after OXT or PLC treatments. This time lapse was used because, within that time limit, the peptide will increase its concentrations within the cerebrospinal fluid. Of the total number of participants, 21 female and 15 male subjects were treated with OXT. The remaining 49 subjects received PLC treatment. During post-experiment interviews, subjects could not identify (with any better degree of accuracy than by chance) whether they had received the OXT or PLC treatment.

### Experimental design

**Figure 1:**
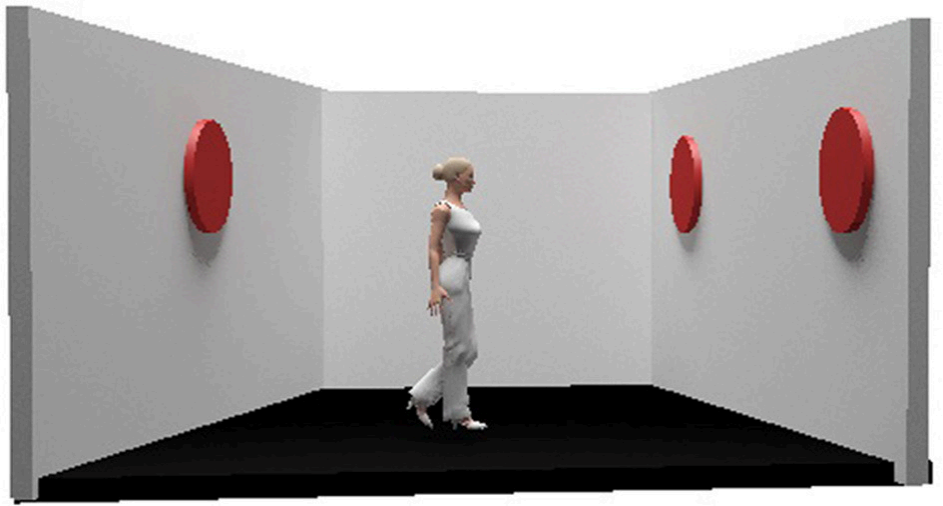
Example of the visual stimulus used for the experiment.

The participants were then presented with a picture showing a lateral view into a room with the left, back, and right walls visible and with red discs displayed on one or two of the walls. Female and male avatars were created with the 3D cartoon software Poser 6 (e frontier, Scotts Valley, California, USA), and were positioned in the center of the room, facing either the left or the right wall. On either side of the room or on opposite sides of the wall, either 0, 1, 2, or 3 red discs were displayed randomly (Figure 1). During the experiment, female subjects were presented with female model avatars and male subjects were presented with male model avatars. In 50% of the experimental sequences, the number of red discs seen by the avatar and the subjects was identical (consistent condition). In the remaining 50% of the experimental sequences, the avatar was positioned in such a way that he or she could not see some of the discs that were visible to the participants (inconsistent condition). In both conditions, the position of the discs changed while the position of the avatar remained constant.

**Figure 2:**
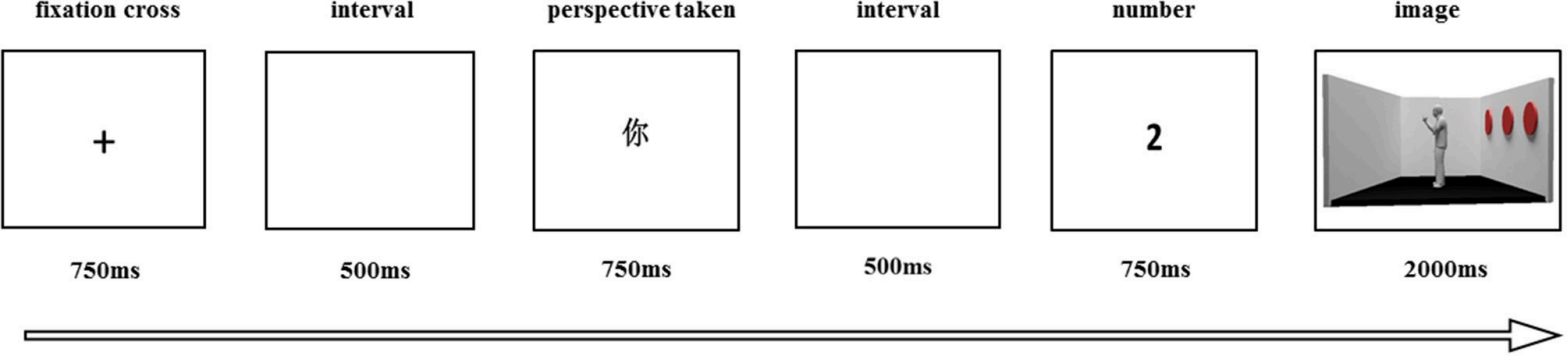
Example trial used for the experiment.

The experiment was controlled by the E-Prime program. At the beginning of the experiment, subjects were familiarized with the process of the task and how to respond to the cues. Each stimulus sequence consisted of four stages (see Figure 2). The stages were as follows: First, a fixation cross appeared for a duration of 750 ms. Second, after an interval of 500 ms, Chinese characters “you” or “him/she?” (male/female avatar) appeared for 750 ms to prompt participants whether to adopt their own perspective (self-condition) or that of the avatar (other condition). After another 500 ms interval, a number ranging between 0 and 3 was displayed, lasting for 750 ms, which specified the number of discs the subject was required to judge. Finally, the image of the room appeared until participants reacted with a the “yes” (matched) or “no” (mismatched) on the keyboard from the given perspective, then went to the next sequence. If the subjects still remained unresponsive at 2000 ms, the next trial appeared automatically.

The experiment included a total of 208 trials, 104 of which required “yes” responses and the 104 remaining stimuli required “no” responses. In the trials of 104 “yes” responses, 48 stimulus trials required the subjects to verify their own perspective (including 24 consistent trials and 24 inconsistent trials) and 48 trials the avatar's perspective (with 24 consistent perspective trials and 24 inconsistent perspective trials). There was an equal number of mismatching (“no”) responses. The experiment also added 16 filler trials where no discs were displayed on the wall. Therefore, the answer “0” was sometimes the correct response. These filler trials included an equal number of self and other trials, consistent and inconsistent trials and “yes” and “no” trials. The experiment was divided into four blocks, each with 52 test trials (48 test trials and four filler tests). Prior to the formal experiment, 26 practice trials were presented. Within each block, the sequence of tests was pseudo-random, then fixed across participants so that there were no more than three consecutive trials of the same type and self and other trials were equally preceded by the same perspective (no shift of perspective) and by a different perspective (shift of perspective). The order of presentation of the blocks was counterbalanced across participants.

Our results were analyzed via SPSS 16.0. Some principles in the analyses of variance have been reported below. When the sphericity hypothesis was violated, we used Greenhouse-Geisser corrections. When follow-up tests were required, Bonferroni corrections were applied.

## Results

We performed a 2 × 2 repeated measure analysis of variance (ANOVA) with treatment type (OXT vs. PLC), gender (male vs. female) as between-subjects factors and the type of perspective taken (self vs. other) and the reaction condition (consistent vs. inconsistent) as within subject variables. Response time and accuracy were used as dependent variables (Table 1).

**Table 1:** The mean and standard deviation of the reaction time and accuracy between male and female subjects at different perspectives and different reaction conditions in both OXT and PLC groups.

		**RT (ms)**				**ACC (%)**			
		**OXT**		**PLC**		**OXT**		**PLC**	
		**Male**	**Female**	**Male**	**Female**	**Male**	**Female**	**Male**	**Female**
Self	Inconsistent	737.72 (34.24)	751.54 (28.94)	663.09 (27.07)	758.97 (26.52)	93.72 (1.53)	94.88 (1.30)	92.50 (1.14)	94.46 (1.18)
	Consistent	722.79 (33.02)	736.96 (27.90)	640.36 (26.10)	723.74 (25.57)	94.74 (1.04)	95.46 (0.88)	94.95 (0.82)	96.92 (0.81)
Other	Inconsistent	771.58 (33.06)	767.29 (27.94)	699.76 (26.13)	808.66 (25.61)	89.40 (1.59)	90.48 (1.35)	90.12 (1.30)	92.00 (1.23)
	Consistent	721.29 (32.74)	731.01 (26.67)	657.26 (25.89)	736.40 (25.36)	94.41 (1.05)	96.57 (0.88)	95.51 (0.83)	95.92 (0.81)

### Reaction time analysis

**Figure 3:**
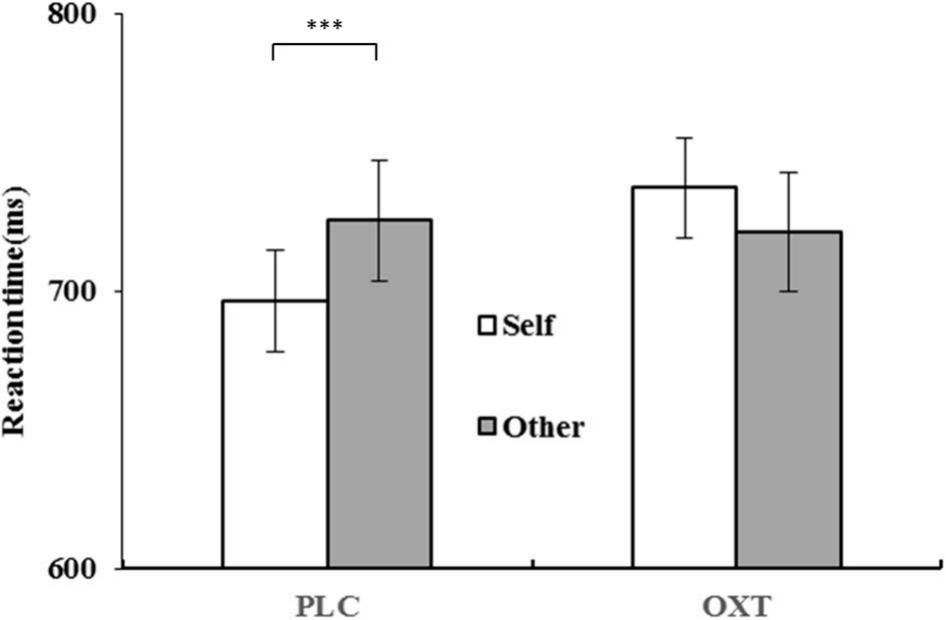
Mean (and standard error) reaction times of perspective taken (self vs. other) in OXT and PLC groups (^***^p < 0.001).

The ANOVA analysis revealed a significant primary effect of reaction condition [*F*_(1, 81)_ = 87.68, *p* < 0.001, η^2^_*p*_ = 0.52] with RTs being overall slower in the inconsistent condition (*M* = 744.83 ms) when compared to the consistent condition (*M* = 702.73 ms). The main effect of perspective taking was also significant [*F*_(1, 81)_ = 21.92, *p* < 0.001, η^2^_*p*_ = 0.21]; participants were significantly quicker when judging from their own perspective (*M* = 717.90 ms) than the avatar's perspective (*M* = 736.66 ms). There was a significant reaction condition × perspective interaction effect [*F*_(1, 81)_ = 40.04, *p* < 0.001, η^2^_*p*_ = 0.33]; a simple effect test showed that under the condition of inconsistency, the judgment of the self-perspective was significantly faster than the judgment of other people's perspective; however, no effect was noted under the consistent condition. We also found the interaction effect between treatment and perspective, *F*_(1, 81)_ = 23.63, *p* < 0.001, η^2^_*p*_ = 0.23, with participants responding faster in the self-perspective judgments (*M* = 696.54 ms) than in other-perspective judgments (*M* = 725.52 ms) in the PLC group; however, no effect was found in the OXT group (Figure 3).

**Figure 4:**
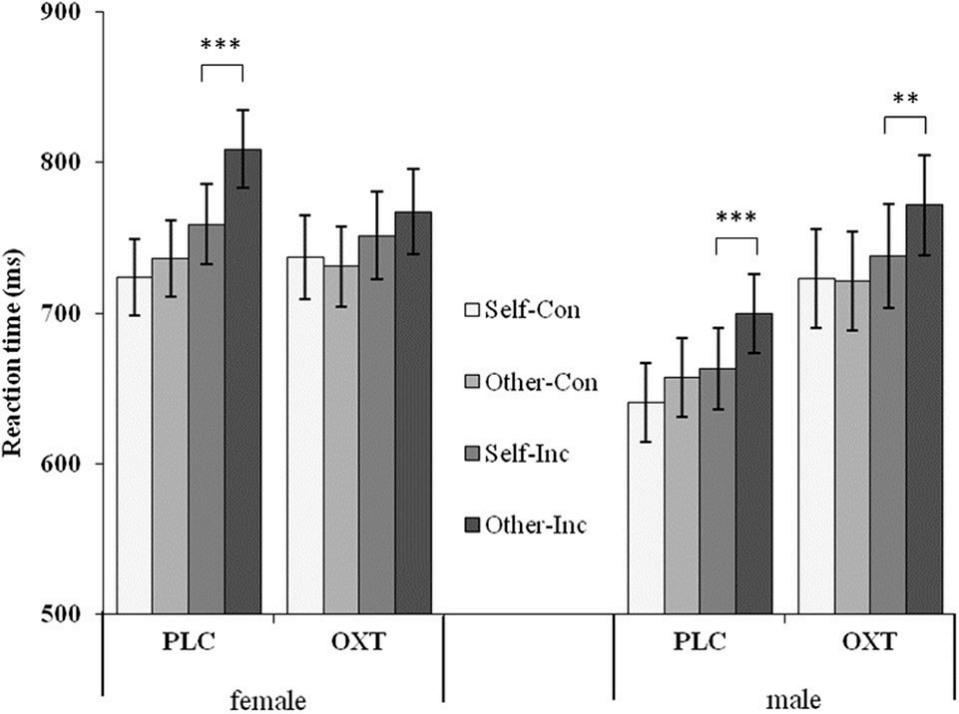
Mean (and standard error) reaction time between male and female subjects from different perspectives and different reaction conditions in both OXT and PLC groups. Symbols indicate significance level (^***^p < 0.001; ^**^p < 0.01).

We were most interested to investigate whether OXT could affect the egocentric biases in the visual perspective taking in response to sex differences; thus, the simple effect test of the perspective taken with the other three factors fixed (treatment, gender, and reaction condition) were performed. The analysis results showed that: (1) For female subjects in the PLC group, participants responded faster in the self-perspective than in the other-perspective under the condition of inconsistency [*F*_(1, 81)_ = 26.20, *p* < 0.001, η^2^_*p*_ = 0.24]; however, they showed no effect in the consistent condition between both perspectives; in the OXT group, no effect was found in the two conditions between the two perspectives. (2) For male subjects, there was a significant perspective effect on both the OXT group [*F*_(1, 81)_ = 7.30, *p* < 0.01, η^2^_*p*_ = 0.08] and the PLC group [*F*_(1, 81)_ = 13.70, *p* < 0.001, η^2^_*p*_ = 0.15] under the condition of inconsistency, with participants being quicker at judging their own perspective than that of the avatar. No effect was found under the consistent condition in both groups between the perspectives (Figure 4).

To examine the effects of OXT on the response time of four experimental conditions, we analyzed the data for male and female participants separately. However, there were no differences between the drug effects on all four conditions for both males and females. I.e., the effects of OXT on the response time of visual perspective taking have not reached the significance level. Then, we analyzed the sex differences in the four experimental conditions for males and females. The results also showed that there were significant gender effects on all four conditions within the subject experimental condition in the PLC group (consistent-self [*F*_(1, 81)_ = 5.21, *p* < 0.05, η^2^_*p*_ = 0.06], consistent-other [*F*_(1, 81)_ = 4.77, *p* < 0.05, η^2^_*p*_ = 0.06], inconsistent-self [*F*_(1, 81)_ = 6.40, *p* < 0.05, η^2^_*p*_ = 0.07], inconsistent-other [*F*_(1, 81)_ = 8.86, *p* < 0.01, η^2^_*p*_ = 0.10], respectively), with males responding faster than females. However, no gender differences were found in all conditions in the OXT group. Based on these results, we concluded that OXT did not lead to a statistically different reaction time of male participants compared to female participants.

### Accuracy analysis

The analysis revealed a significant main effect on reaction condition [*F*_(1, 81)_ = 57.14, *p* < 0.001, η^2^_*p*_ = 0.41] with less accuracy when inconsistent (*M* = 92.17%) than when consistent (*M* = 95.56%). The main effect of perspective was also significant [*F*_(1, 81)_ = 12.44, *p* = 0.001, η^2^_*p*_ = 0.13]; participants were significantly more accurate when judging from their own perspective (*M* = 94.60%) than when judging from the avatar's perspective (*M* = 93.13%). There was a significant treatment × reaction condition × perspective interaction effect [*F*_(1, 81)_ = 5.26, *p* < 0.05, η^2^_*p*_ = 0.06]. A further simple effect test showed that, under the condition of inconsistency on both OXT and PLC groups, the accuracy was higher in the self-perspective judgments (*M*_PLC_ = 93.06%, *M*_OXT_ = 94.30%) than other-perspective judgments (*M*_PLC_ = 91.39%, *M*_OXT_ = 89.94%). However, there was no effect under the consistent condition between the two perspectives on both OXT and PLC groups.

**Figure 5:**
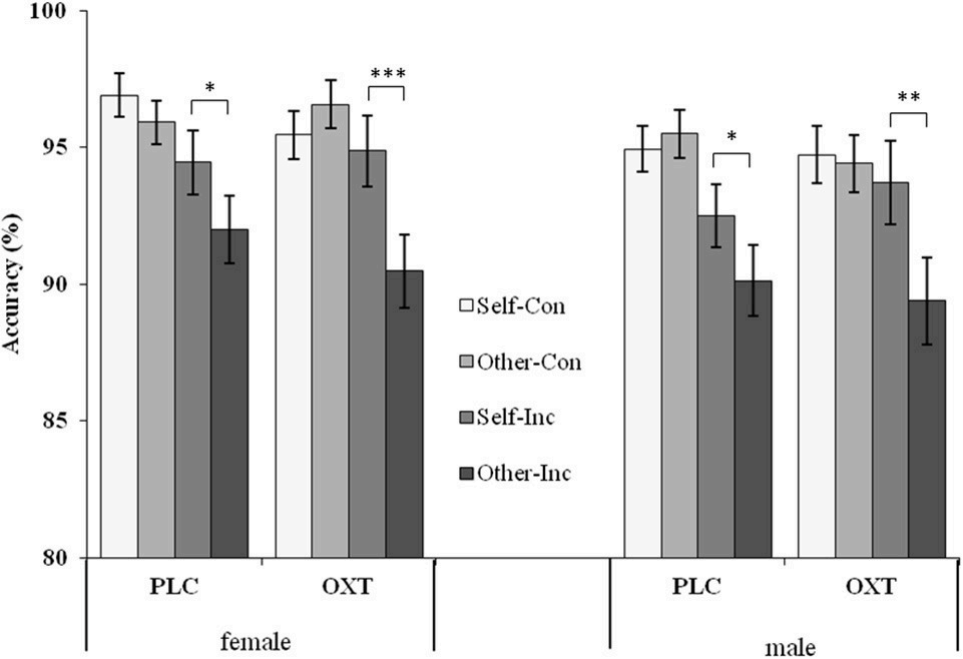
Mean (and standard error) accuracy between male and female subjects at different perspectives and different reaction conditions in both OXT and PLC groups. Symbols indicate significance level (^***^p < 0.001; ^**^p < 0.01; ^*^p < 0.05).

Further ANOVAs were performed for males and females separately to elucidate the effects of OXT on visual perspective taking. The results showed that, under the condition of consistency, there was no significant difference between the two perspectives in both females and males. However, judging from their own perspective always yielded an ACC advantage when compared to judging from the avatar's perspective under the inconsistent condition in both females and males in the two groups [*F*_female−PLC(1, 81)_ = 4.44, *p* < 0.05, η^2^_*p*_ = 0.05; *F*_female−OXT(1, 81)_ = 11.94, *p* = 0.001, η^2^_*p*_ = 0.13; *F*_male−PLC(1, 81)_ = 4.28, *p* < 0.05, η^2^_*p*_ = 0.05; *F*_male−OXT(1, 81)_ = 8.79, *p* < 0.01, η^2^_*p*_ = 0.10] (see Figure 5). Therefore, the egocentric biases performed in the consistency condition were not affected by OXT.

Corresponding to the analysis of reaction time, we analyzed the effects of OXT and sex for four experimental conditions separately. With regard to the effect of OXT, there were no significant differences in all four subject experimental conditions neither in males nor in females. Furthermore, no sex differences existed in all the conditions regardless of group (OXT group or PLC group). It seemed that OXT had no effect on the accuracy of participants.

## Discussion

In this study, we investigated the different effects of OXT on visual perspective taking for men and women. The results showed that OXT reduced differences in reaction time between judging from one's own perspective and judging others' perspectives in female participants under the inconsistent condition. In contrast to females, male participants who received intranasal OXT still showed a significantly slower reaction time when taking on the perspective of another compared to their own perspective, independent of whether the reaction condition was consistent or inconsistent; however, OXT yielded similar reaction times in males than in females across all four experimental conditions. With regard to accuracy, male and female participants were significantly less accurate when taking on the perspective of another compared to their own perspective in the inconsistent condition, which was true for both drug conditions.

The results of the PLC group validated the normal state performance in the visual perspective taking task of previous studies. Firstly, egocentric biases were also present in our PLC group. More precisely, under the consistent condition, there were no significant differences in response time and accuracy between the participants' own perspective and the avatar's perspective; however, under the condition of inconsistency, self-perspective judgments had a significant advantage for both response time and accuracy when compared to other-perspective judgments. These results indicate that in the reasoning process of others' mental states, the information from the perspective of the self plays an important role, which is consistent with a considerable body of previous research (Keysar et al., 2003; Bernstein et al., 2004; Birch and Bloom, 2007; Apperly et al., 2009). This might be because people tend to anchor from their own point of view and then only adjust from self-perspective to other-perspective, overcoming self-centeredness to arrive at a final judgment (Epley et al., 2004). Thus, under the inconsistent condition, the participants were thrown off by varying information in the self-perspective when judging from the avatar's perspective than when compared with the consistent condition, in which they required more time to adjust and correct egocentric bias. Secondly, we also observed the sex differences in performance in the PLC group. Although the differences between males and females were absent for participants' accuracy, the responses of male subjects were significantly faster than those of female subjects throughout all experimental conditions. Our results were in line with a previous relevant study by Mohr et al. (2010), which showed that women experienced increased reaction times compared to men when performing an avatar perspective task. Mohr et al. (2010) suggested that these results may be caused by a difference in processing strategies between men and women: the object-based spatial strategies may be more prevalent in men, which renders them good at spatial/mental rotation and enables them to spent less time on such tasks; however, women may be inclined to adopt a social perspective taking strategy, which is supported by the link between high empathy and faster reaction times; therefore, this strategy is comparatively more time-consuming when they finish the task.

Partially consistent with our hypothesis, we found no significant differences between the two perspectives in response time after OXT administration in female participants, regardless whether the condition was consistency or inconsistency. Compared to the results of the PLC group, it seems that OXT shortened the reaction time difference between self- vs other-perspective taking in the inconsistent condition. According to the previous discussion, the results may indicate that OXT has the potential to allow female participants to effectively avoid the interference of their own perspective when judging from the avatar's perspective, which leads to a more rapid reaction. This is in line with OXT's function reported in previous studies; i.e., a shift in focus from self to others (Abu-Akel et al., 2015; Bartz et al., 2015). In general, the results of this study support our hypothesis: OXT administration may decrease self-centeredness and increase focus in others, which renders female participants quicker in inferring the mental state of others. However, the results did not show that OXT has an effect on accuracy between both perspectives. Specifically, the participants' information from their self-perspectives still influenced the process of adopting the views of the avatars, lending more accuracy when judging from one's own perspective than from others' perspectives. The results agree with similar results and explanations provided by other studies; i.e., Hubble et al. (2017) and Di Simplicio et al. (2009), who also reported a lack of effects of OXT on the accuracy in their tasks. This may indicate that the weakening effect of OXT on the egocentric bias in perspective taking may also have limitations, which indicates that self-centeredness is, to a great degree, still a default choice when accurately inferring other people's mental states. However, the behavioral tasks may be less sensitive to OXT triggered changes or just too low in difficulty. Indeed, in our results show that the accuracy was very close to 100%, even if men would start thinking harder in this task, there is almost no room left to improve. Thus, although we found that OXT could enhance individual's attention to others' perspectives and accelerate the process of suppression and correction of egocentric tendencies, future studies are required to further explore the effects of OXT in the process of perspective taking.

Another important foundation of this research was the differential effect of OXT on visual perspective taking between men and women. It seemed that OXT had no effect on male egocentric tendencies in the visual perspective taking task, because they were still advantageous with significantly quicker reaction time and higher accuracy when taking on their own perspective compared to that of the avatar in the inconsistent condition. However, after inhaling OXT, the differences in the response time between sexes on all experimental conditions disappeared; however, it still existed for the accuracy index. The following two reasons can explain the reducing effect of OXT on the response time between males and females: Firstly, we noticed that the response time of OXT group had an accelerated trend in females compared to the PLC group; however, this was not significant (756.94 ms vs. 746.70 ms). The effects of OXT on promoting the empathy ability in women has been verified in the study of Mohr et al. (2010), who reported that women with higher empathy scores responded faster in the perspective taking task. Secondly, OXT led to a trend of reducing the response time in men compared to the PLC group (665.12 ms vs. 738.35 ms; also not significant). This result is consistent with the results of Theodoridou et al. (2013), which showed that male participants in the OXT group responded as slowly as females. According to the explanation by Theodoridou et al. (2013), this may indicate that OXT promotes the attempts of male subjects to adopt similar perspective taking processing strategies as female subjects, and as such, there is a slowing trend in response time.

The question remains why differential effects of OXT on visual perspective taking for men and women still exist. Unfortunately, no definite explanation exists up to now. As far as our research is concerned, this difference may be caused by a variety of factors. Firstly, females are better at taking on the views of others compared to males. While OXT could increase both male and female perspective taking, women may be more affected than men, and thus easier to remain in their initial behaviors. In addition, previous studies found that steroid hormones, such as estradiol and progesterone, can modulate the OXT receptor (Gimpl and Fahrenholz, 2002; Choleris et al., 2008). Essentially, women differ from men with regard to gonadal steroid hormones (Hawkins and Matzuk, 2008). Therefore, the modulation of OXT by gonadal steroids, which affects the differences in the sensitivity to the OXT system, might be an explanation for the inconsistent findings (between men and women) in our tasks.

## Limitations

This study has several limitations, which can be addressed in future studies. First, although the results showed that women are likely to be more sensitive to OXT than men, the results were concluded based on a relatively small sample size. Comparing male and female performance in larger samples is thus necessary to draw definite conclusions about differences between sexes. Next, the task in our study may be too simple for participants and the ceiling effects appeared to make our results very complicated; i.e., the used task was not suited to detect performance improvements due to OXT. Thus, future studies should overcome this insufficiency using a better experiment task. Then, we used a between-subjects design for drug administration, while individual differences, such as psychological factors, may also moderate the effects of OXT (Daughters et al., 2015). Controlling for these variables may advance our understanding of the OXT's effect in visual perspective taking in future studies. Ultimately, the behavioral indicators we used in this study (response time and accuracy), may be insensitive to the changes induced by OXT, thus future researcher should select more sensitive indicators, such as event-related potentials studies, to further explore this topic.

## Conclusion

In summary, this study investigated the effects of OXT on men and women in a visual perspective taking task. The results showed that OXT shortened the differences of response time between men and women regardless of whether they were taking the perspective of self or others. In addition, after OXT administration, the difference in reaction time between judging from one's own perspective and judging from others' perspectives also decreased in female participants, but this effect was not present in male participants. The above results may indicate that OXT could increase perspective taking abilities through reducing self-bias and increasing the perception of others and this trend is mainly reflected in women rather than in men.

## Author contributions

TY and XH designed experiments; TY, YJ, and CY carried out experiments. TY analyzed sequencing data and wrote the manuscript.

### Conflict of interest statement

The authors declare that the research was conducted in the absence of any commercial or financial relationships that could be construed as a potential conflict of interest.
